# Ultrasound Risk Stratification of Autonomously Functioning Thyroid Nodules: Cine Loop Video Sequences Versus Static Image Captures

**DOI:** 10.3390/diagnostics15192525

**Published:** 2025-10-06

**Authors:** Larissa Rosenbaum, Martin Freesmeyer, Tabea Nikola Schmidt, Christian Kühnel, Falk Gühne, Philipp Seifert

**Affiliations:** Clinic of Nuclear Medicine, Jena University Hospital, Am Klinikum 1, 07747 Jena, Germany; larissa.rosenbaum@med.uni-jena.de (L.R.); philipp.seifert@med.uni-jena.de (P.S.)

**Keywords:** thyroid, ultrasound, cine loop, TIRADS, scintigraphy, autonomously functioning, thyroid nodules

## Abstract

**Background/Objectives**: Autonomously functioning thyroid nodules (AFTNs) are most frequently diagnosed as benign. However, they show high ratings in ultrasound (US) risk stratification systems (RSSs) that utilize the current clinical standard methodology of conventional static image capture (SIC) documentation. The objective of this study was to evaluate the RSS ratings and respective fine needle cytology (FNC) recommendations of cine loop (CL) video sequences in comparison to SIC. **Methods**: 407 patients with 424 AFTNs were enrolled in this unicentric, retrospective study between 11/2015 and 11/2023. Recorded US CL and SIC were analyzed lesion-wise and compared regarding US features, Kwak and ACR TIRADS, ACR FNC recommendations, as well as assessment difficulties and artifacts. Statistical analyses were conducted using the Chi^2^ test and Spearman’s correlation coefficient in SPSS software. *p*-values < 0.05 were considered significant. **Results**: Strong to very strong correlations were observed for all US features, RSS ratings, and ACR FNC recommendations (Spearman’s correlation: each *p* < 0.001), comparing CL and SIC. For >60% of the AFTNs, ACR FNC recommendation was given. Kwak TIRADS were more consistent with the benign nature of AFTNs than the ACR ratings. CL captured significantly more “echogenic foci” than SIC (Chi^2^: *p* < 0.001). Artifacts (poor image quality, acoustic shadowing, sagittal incompletely displayed AFTN) were significantly more common on CL, affecting ~40% of AFTNs, compared to ~15% on SIC (Chi^2^: each *p* < 0.05). Weak correlation was observed for assessment confidence between CL and SIC, with SIC outperforming CL (Spearman’s correlation: each *p* < 0.001). **Conclusions**: A strong correlation was identified between CL and SIC in terms of RSS ratings and ACR FNC recommendations. Kwak is a superior representative of the benign character of AFTNs than ACR. However, CL provided more detailed information while being associated with decreased observer confidence and more artifacts. Specific operator training and technical improvements, including AI implementation, could improve image quality in future.

## 1. Introduction

Thyroid nodules (TNs) are a prevalent clinical finding, with a significant number being classified as benign [1]. In iodine-deficient regions, such as Germany, the increasing incidence of nodules necessitates a comprehensive understanding of their diagnostic evaluation [2]. Thyroid ultrasound (US) is the basic imaging method for the assessment of the thyroid gland [3,4]. For detected nodules, risk stratification systems (RSSs) such as the Thyroid Imaging Reporting and Data System (TIRADS) are used to classify their dignity [5]. RSSs are helpful in standardizing the assessment of TNs and supporting precise differentiation between benign and malign lesions [6]. One purpose is to enable unexperienced users to achieve reliable results. In a previous study, high diagnostic accuracies were achieved by a novice observer after a short training interval [7].

Autonomously functioning thyroid nodules (AFTNs) are considered to have a very low risk of malignancy [8]. However, research has demonstrated that a relevant proportion of AFTNs manifest high-risk categories in RSS analyses, a phenomenon that is particularly evident in regions experiencing iodine deficiency [9,10]. In light of these findings, scintigraphy is a valuable tool for the risk stratification of thyroid nodules [11].

In the clinical routine, Tc-99m-pertechnetate is used for thyroid scintigraphy, allowing for a functional evaluation of TNs. The ability of the radiotracer to mimic iodide, the substrate for thyroid hormone synthesis, allows for it to be actively transported into thyroid follicular cells via the sodium iodide symporter. It is not further metabolized there, making it a valuable tool for assessing the functional status of the thyroid. AFTNs appear as hyperfunctioning (“hot”) TNs due to their increased and independent uptake of Tc-99m-pertechnetate [12].

Thyroid-stimulating hormone (TSH), thyroxin (T_3_), and triiodothyronine (T_4_) are the key biochemical parameters used to assess the hormonal status of the thyroid. A suppressed TSH level is an important indicator for hyperthyroidism, while T_3_ and T_4_ levels may remain within the normal range or can be elevated [13]. Due of the hormonal excess, patients can manifest a variety of clinical symptoms such as weight loss, heat intolerance, palpitations, tremor, anxiety, hair loss, and a goiter of the thyroid gland [14].

The standard for thyroid ultrasound documentation remains the recording of static image captures (SICs) alone. This approach precludes the possibility of a comprehensive review of the entire organ. Consequently, important aspects of individual TNs or thyroid morphology in general may be overlooked and cannot be recalled. Since 2015, the Clinic of Nuclear Medicine at the University Hospital in Jena (Germany) has established the recording of cine loop (CL) video sequences of the thyroid in sagittal and transverse planes according to a specific acquisition Standard Operating Procedure (SOP) [15]. The entire thyroid including all TNs can be captured in sectional image series and stored to a local Picture Archiving and Communication System (PACS). This approach facilitates second readings, precise follow-up assessments, and education for novices and students. Additionally, the processes of acquiring CL and storing them in the local PACS can be carried out by non-physician personnel [16].

The role of CL for the assessment of TNs has been the subject of a limited number of recent publications. A study conducted by Seifert et al. found that CL allows for reliable second reading results and can be applied by non-physicians [16]. A substantial degree of interobserver variability was identified, which is likely attributable to varying levels of experience and training among observers [17]. Schmidt et al. demonstrated high diagnostic accuracies of a novice rater for cytopathologically and/or histopathologically diagnosed TNs using TIRADS on CL [7].

However, to the best of our knowledge, using CL for the risk stratification of AFTNs has not been reported yet. To build on the previous findings, this study aims to further explore the potentials and limitations of CL in thyroid diagnostics by expanding the existing research and focusing on AFTNs.

## 2. Materials and Methods

### 2.1. Study Population

This retrospective unicentric study enrolled all consecutive patients with diagnosed AFTNs from the Clinic of Nuclear Medicine at the University Hospital in Jena (Germany) between November 2015 and November 2023. Prior to participation, all patients provided written consent for the anonymized use of their data for research purposes and subsequent publication. This consent was formalized through the signature of an admission to hospital form (“Aufnahmeantrag”). No age-range limitation was imposed on the study participants.

Following inclusion criteria were applied: (1) adult patients with unifocal, bifocal, or trifocal AFTNs on Tc-99m-pertechnetate scintigraphy (TS); (2) unambiguous correlation between lesions on TS and US; (3) US CL and SIC of every included AFTN available on PACS; (4) use of GE LOGIQ E9 US device.

**Figure 1:**
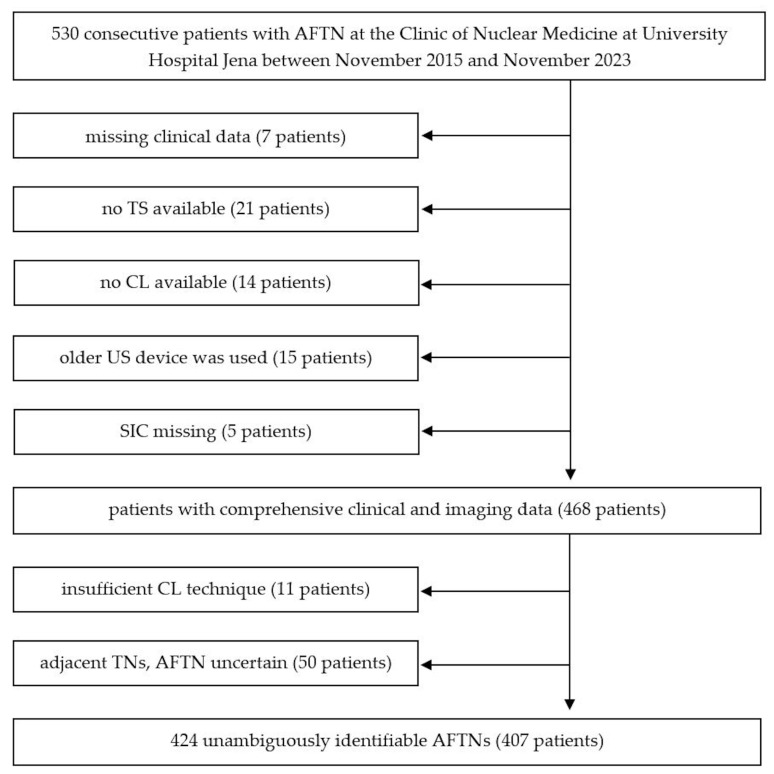
Flowchart of the study participants. Abbreviations: AFTN—autonomously functioning thyroid nodule; TS—Tc-99m-pertechnetate scintigraphy; SIC—static image captures; CL—cine loops; US—ultrasound; TN—thyroid nodule.

Patients were excluded from the study if (1) relevant clinical data were missing; (2) no CL or TS images were available (preliminary examinations were externally performed, AFTNs were only described in the medical reports); (3) an older US device was used; (4) SIC were missing; (5) several TNs were closely adjacent and the AFTN (depicted on TS) was not clearly definable; and (6) CL technique was insufficient (e.g., missing sagittal US scans, >50% of AFTN not shown on any CL). An overview of the patient selection is shown in Figure 1.

### 2.2. Thyroid Ultrasound and Scintigraphy Examinations

The sonographic examinations were performed by 25 different physicians in clinical routine using the GE LOGIQ E9 XDclear 2.0 US device equipped with a linear ML6-15 probe (GE Healthcare, Milwaukee, WI, USA). The additional documentation of CL, according to a local SOP, was part of the local clinical standard of care [7,15,16]. The clinically indicated planar TSs were performed in accordance with the German guidelines using Tc-99m-pertechnetate tracer and the Nucline TH22 gamma camera (Mediso, Budapest, Hungary) [18].

### 2.3. Data Assessment and Observer

Clinical data contained age, sex, laboratory thyroid parameters, (pre)medication, pretreatment (surgery, radioiodine therapy), iodine exposition, thyroid gland volume, thyroid tracer uptake, and comprehensive AFTN data (see below). The reference range for TSH was defined according to the standards of the local hospital (0.25–4.04 mU/L). Elevated TSH levels (>4.04 mU/L) were indicative of hypothyroidism, whereas decreased TSH levels (<0.25 mU/L) indicated hyperthyroidism.

A single novice observer (medical student, 3rd academic year) underwent a 1 h educational teaching by an experienced TIRADS expert (senior author of this study). The training curriculum encompassed all the essential skills required for the evaluation of TNs. These included the assessment of US features and the application of TIRADS. The short curriculum also covered the technical background of US in general and CLs in particular. In addition, comprehensive self-studying of approximately 15 h was carried out. An extensive literature review was conducted, encompassing the most pertinent and recent original studies, meta-analyses, reviews, and guidelines. This extensive review provided a solid knowledge foundation before initiating the image analysis [19,20,21,22,23,24,25,26,27,28]. In addition to the training and self-studying, *n* = 20 training TNs were jointly reviewed alongside the experienced physician. This collaborative approach continued throughout the data analysis. Any AFTN with uncertainties was flagged and then reviewed with the TIRADS expert senior physician (*n*~30).

For the assessment of the AFTNs, following aspects were considered: location, diameter, volume, individual US features, risk stratification according to Kwak, and American College of Radiology (ACR) TIRADS [29,30]. The overall level of confidence in the assessment was documented using a 4-point scale (very confident, confident, unsure, and very unsure), and the documentation of the most significant uncertainty factor was conducted at the level of each lesion.

Moreover, technical US details such as artifacts, number of foci and frames, foci and frames covering AFTN and full representation of ATFN on images were documented. These details were identified and documented by the observer during the TIRADS assessment. Artifacts were defined as poor image quality, acoustic shadowing, and incompletely displayed AFTNs. The assessment of the image quality was focused on the target lesions (AFTNs) and included blurring and unsharp images due to inadequate US device settings like focus positioning, frequency, or depth. The phenomenon of acoustic shadowing was identified by the presence of anechoic areas superimposed to the ATFNs. Acoustic shadowing was mainly caused by macro- or peripheral (rim) calcifications, and other reasons like insufficient US gel application. All artifacts were separately documented in transverse and sagittal orientation. When a AFTN was incompletely displayed, the cut proportion was documented.

Initially, the review of CL was conducted, subsequently followed by an evaluation of SIC. A 3-month interval was maintained between the two assessments to mitigate the influence of recognition bias. The novice observer was aware of the functional characteristics of the lesions and was not blinded to the diagnosis AFTN.

### 2.4. Data Analysis and Statistics

All data were recorded on Microsoft Excel software (Microsoft Corporation, Version 2016, Redmond, WA, USA). Statistical tests were performed using IBM SPSS Statistics software (International Business Machines Corporation, Version 30.0, New York, NY, USA).

The nominal and ordinal variables were tested using the Chi2 test. *p*-values < 0.05 were considered statistically significant, indicating significant differences between the compared subgroups and leading to the rejection of the null hypothesis.

To examine correlation among SIC and CL, Spearman’s correlation coefficients (r_s_) were calculated. A significant correlation was considered if the *p*-value was < 0.05, thereby indicating that the observed association between the two variables was not the result of random chance or measurement errors. The null hypothesis was rejected in these cases. An established scale for Spearman’s correlation coefficients was utilized to define the strength of relationship between CL and SIC: 0.299 or less represented none to poor agreement, 0.3–0.399 indicated moderate agreement, 0.4–0.699 strong agreement, 0.7–0.999 very strong agreement, and 1.0 signified perfect correlation [31]. To also determine Spearman’s correlation coefficient for AFTNs with multiple echogenic foci, the aggregate sum of ACR TIRADS points for all documented foci in this particular lesion was considered [29].

The subgroup “none or large comet-tail artifacts” of the US feature “composition” was subdivided in 2 separated groups: “none” and “large comet-tail artifacts”.

For ACR TIRADS, TR4 was further classified into TR4 (4 pts.), TR4 (5 pts.), and TR4 (6 pts.) [27]. Likewise, in Kwak TIRADS, 4C was subdivided into 4C (3 pts.) and 4C (4 pts.) [7].

## 3. Results

### 3.1. Patient Characteristics and Clinical Data

A total number of 407 adult patients with 424 identified AFTNs were included in this study. The majority of the patients exhibited hyperthyroidism at the time of TS and revealed enlarged thyroid organ volumes. A Tc-99m-pertechnetate uptake of 1.9 ± 2.4% (mean: 1.5%, range: 0.44–37.0%) was observed. The measured largest diameters showed no significant difference between CL and SIC (*p* = 0.555). Table 1 provides detailed information.

### 3.2. Ultrasound Features

The majority of the assessed US features displayed strong correlation between CL and SIC, including echogenicity, shape, margin, and echogenic foci (r > 0.400, *p* < 0.001). For composition, a very strong correlation was identified (CL versus SIC) (r > 0.700, *p* < 0.001). Significant differences for the subgroups “none”, “large comet-tail artifacts”, and “macrocalcifications” of echogenic foci were found (CL versus SIC). Detailed information is presented in Table 2.

### 3.3. Risk Stratification According to TIRADS

Strong correlation between CL and SIC of Kwak and ACR TIRADS were observed (r > 0.400, *p* < 0.001), but for both investigated RSS no significant difference was found. Fine-needle cytology (FNA) was recommended for the majority of AFTNs, both for CL (67.9%) and SIC (63.9%), with a strong correlation (r > 0.400, *p* < 0.001) and no significant differences. Table 3 summarizes the results.

### 3.4. Technical Ultrasound Features

During the examination of AFTNs on CL and SIC, a significantly lower number of artifacts was detected on SIC (*p* < 0.001). The presence of acoustic shadowing and poor image quality was found to be significantly more prevalent in CL. AFTNs were more likely to be incompletely visualized on sagittal CL, in comparison to sagittal SIC (*p* < 0.001). Further information is presented in Table 4.

The manual focus setting on the GE LOGIQ E9 US unit was an important factor in the quality of the US images. On SIC, in the transverse (sagittal) US planes, 60.6% (58.1%) of the AFTNs were captured by all existing foci, and 1.5% (2.0%) by none. The number of foci, capturing the AFTNs in transverse (sagittal) planes, ranged from 0 to 3 (0–3) with a mean of 1.6 ± 0.7 (1.7 ± 0.7) and a median of 2 (2). On CL, in the transverse (sagittal) planes, 30.0% (30.0%) of the AFTNs were covered by every existing focus and 0.5% (0.2%) were not covered by any focus. The number of foci covering each AFTN ranged from 0 to 4 (0–3) with a mean of 2.0 ± 0.7 (1.9 ± 0.7) and a median of 2 (2). Thus, a higher number of foci covering AFTNs was observed on SIC in comparison to CL (*p* < 0.001).

The quantity of frames within a CL is strongly related to its quality. Slower motions lead to more frames, resulting in higher image resolution. In the intragroup comparison of CL, in transverse planes, the number of frames ranged from 49 to 490 (mean: 138 ± 66, median: 124), and in sagittal from 27 to 314 (mean: 107 ± 46, median: 99). The number of frames covering AFTN ranged from 6 to 210 (mean: 39 ± 25, median: 33) in transverse and in sagittal from 4 to 183 (mean: 51 ± 28; median: 45).

### 3.5. Assessment Obstacles

Weak correlations were identified for assessment confidence and US features of difficulty between CL and SIC (r < 0.299, *p* < 0.05). All categories, from “very confident” to “very unsure”, showed significant differences (CL versus SIC), revealing a higher overall assessment confidence for SIC. While assessing AFTN, a higher number of challenging US features were documented on CL in comparison to SIC (*p* < 0.001). Composition (*p* = 0.029), margin (*p* < 0.001), and echogenic foci (*p* < 0.001) were less often encountered as obstacles on SIC (CL versus SIC). For a total of 168 AFTNs observed on CL and 303 AFTNs on SIC, no challenging US features were identified, resulting in the confidence rating “very confident”. Further details are displayed in Table 5.

## 4. Discussion

US has emerged as the prevailing imaging modality for the evaluation of TNs [32]. Despite its established role, SIC documentation is inherently subject to constraints, particularly concerning retrospective evaluations [16,17]. The issue of the absence of relevant findings was addressed in 2015 by the establishment of CL in our department, thus ensuring the preservation of complete data on PACS [15,33]. Due to a comprehensive review of thyroid US, CL hold the potential to enable unexperienced users to obtain reliable results in the evaluation of thyroid lesions [7]. Another important advantage of CL is its simplicity and speed of implementation [34]. This allows for the acquisition to be easily performed by non-physicians [16].

In this study, a large cohort of patients diagnosed with AFTNs were evaluated according to Kwak and ACR TIRADS using CL versus SIC [29,30]. Strong correlations between CL and SIC were revealed for both RSSs.

Kwak TIRADS, as an easily applicable RSS, turned out to be more effective in evaluating AFTNs compared to ACR TIRADS; 4.5–5.7% of AFTNs were rated Kwak 4C, whereas 45–50% were classified as ACR TR4 or TR5. Notably, the largest group of AFTNs in our study was classified as ACR TR4 (~35–40%). Kwak 4C was subdivided in 4C (3 pts.) and 4C (4 pts.), and for ACR in TR4 (4 pts.), TR4 (5 pts.), and TR4 (6 pts) [27]. Each additional point could potentially increase the malignancy risk [7,35].

In relation to this particular subdivision of ACR TR4, it could be considered whether to modify the recommendation relating to ACR FNC. Setting the cutoff between benign and malign at TR5, could also improve the effectiveness of ACR TIRADS [36]. It is noteworthy that no AFTN was assigned a 4C (4 pts.) or higher rating according to the Kwak TIRADS classification. This finding suggests that AFTNs may possess characteristics that are less likely to suggest malignancy when evaluated using this system.

**Figure 2:**
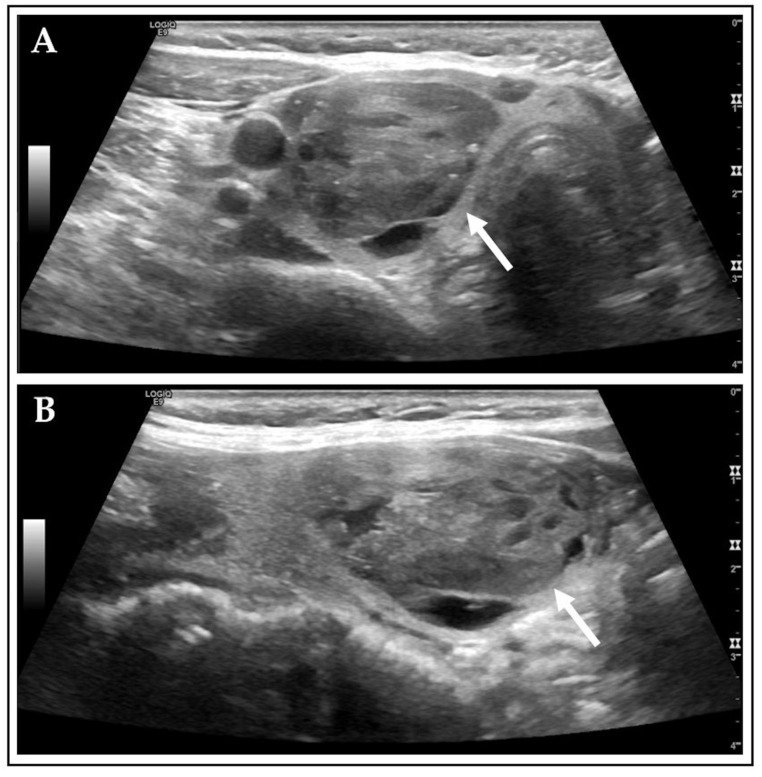
Example of disconcordant Kwak and ACR ratings. Shown is an AFTN on CL image captures, in transverse (**A**) and sagittal (**B**) plane, marked with white arrows, classified as Kwak 4A and as ACR TR4 based on the following US features: mixed cystic and solid (Kwak: 0 pts.; ACR: 1 pt.), hypoechoic (Kwak: 1 pts.; ACR: 2 pts.), wider-than-tall (Kwak and ACR: 0 pts.), smooth margins (Kwak and ACR: 0 pts.), and macrocalcifications (Kwak: 0 pts.; ACR: 1 pt.) [29,30,38].

As demonstrated in Figure 2, an AFTN received a rating of 4 points on the ACR scale, whereas it received only 1 point according to Kwak TIRADS. This reflects the discrepancies between the two investigated RSSs. The inconsistency is attributed to common features of AFTNs, which often comprise a mix of cystic and solid components (>60%) and exhibit a high number of echogenic foci, such as macrocalcifications (>25%) [37]. These characteristics tend to elevate ACR TIRADS scores. In comparison, Schmidt et al. observed ~40% mixed cystic and solid components and ~15% macrocalcifications in cytopathologically and/or histopathologically diagnosed TNs [7].

**Figure 3:**
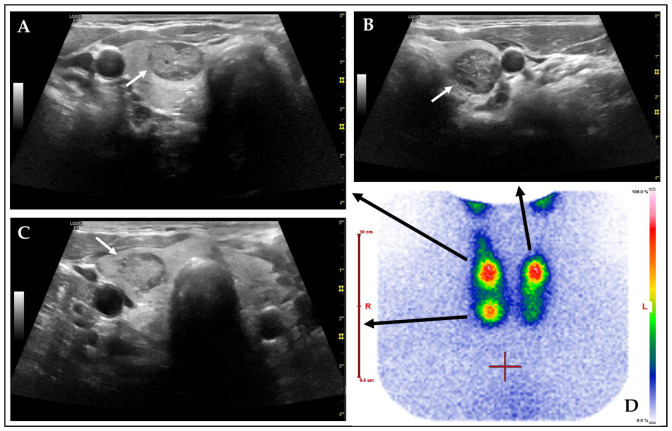
Representative US and Tc-99m-pertechnetate thyroid scintigraphy. Example of a TS with trifocal AFTNs (**D**). Associated TNs (black arrows) are shown on transverse CL US image captures (**A**–**C**, white arrows). (**A**) and (**B**) are located in the right lobe, **C** in the upper left lobe. All AFTNs displayed obtained elevated TIRADS scores with recommendation for ACR FNC. Each of them is exhibiting punctuate echogenic foci.

Previous studies, such as those by Schenke et al., found that 25% of AFTNs, classified using Kwak TIRADS, were rated 4C or higher [9]. Noto et al. and Castellana et al. observed that 31.4–33.3% of AFTNs were classified ACR TR4 or higher [10,39]. None of the aforementioned studies have made a comparison between Kwak and ACR TIRADS. These findings corroborate our study’s observation that a considerable proportion of AFTNs are rated as suspicious, despite the general assumption that AFTNs are almost exclusively benign [40]. This discrepancy can result in recommendations for unnecessary FNC [37,39,41]. In our study, over 60% of AFTNs obtain a recommendation for FNC according to ACR TIRADS due to their score and size. These results highlight the importance of scintigraphy in addition to thyroid US in order to avoid unnecessary FNCs [42]. An example of a TS with associated AFTNs on US is illustrated in Figure 3.

**Figure 4:**
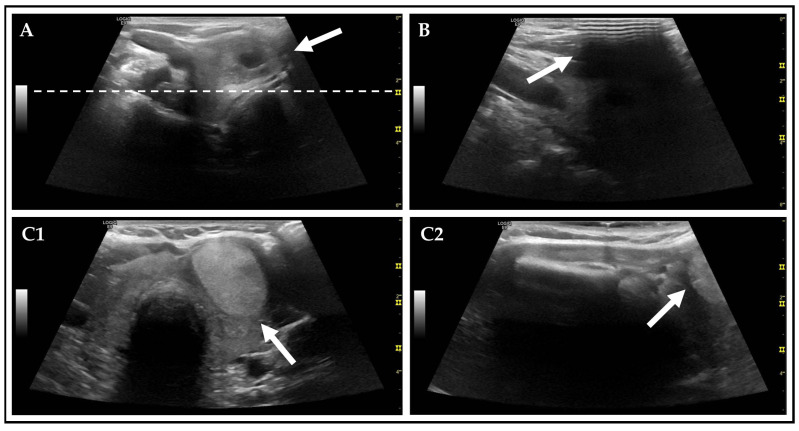
Examples of US artifacts on CL image captures. (**A**): Improper foci setting (white dotted line) for the assessment of the AFTN (white arrow). (**B**): Acoustic shadowing due to inadequate US gel application (white arrow), superimposing an AFTN. (**C1**): Transverse plane with fully captured solid AFTN (white arrow) in the left lobe of the thyroid. (**C2**): Corresponding sagittal plane of the same AFTN (white arrow), located in the lower pole, <50% of AFTN is captured, not allowing cranio-caudal measurement.

It has been observed that CLs are often associated with a significantly higher occurrence of artifacts (~40%) when compared to SIC. These artifacts affect the overall image quality and might be challenging in the assessment of TNs [43,44,45]. For example, blurred images are caused by factors such as movement speed of the US transducer, insufficient frame rates, improper placement of foci, and patient motion due to breathing (Figure 4) [7,16]. Poor image contrast is likely attributable to suboptimal US device settings, such as inappropriate focal depth or frequency, resulting in inadequate visualization of posterior nodule components [46,47].

CL video sequences are recorded in accordance with a standardized SOP, thereby ensuring optimal US video quality for the entire organ [15]. This indicates that the foci settings are not adapted to single lesions, rather encompassing the entire organ as comprehensively as possible. Consequently, a more extensive distribution of foci is necessary. However, in theory, the foci setting on SIC can be adjusted and optimized to the respective TN.

On CL, TNs are significantly more often visualized incompletely on sagittal planes (17%) (CL versus SIC) [16]. Consequently, the cranio-caudal measurement was not feasible in these cases (see Figure 4), and as a result, the recommendation regarding ACR FNC could not be made. This limitation can be prevented by raising awareness among examiners. By ensuring that the sagittal scan is adjusted individually based on the location of the TN, the occurrence of this artifact can be minimized.

**Figure 5:**
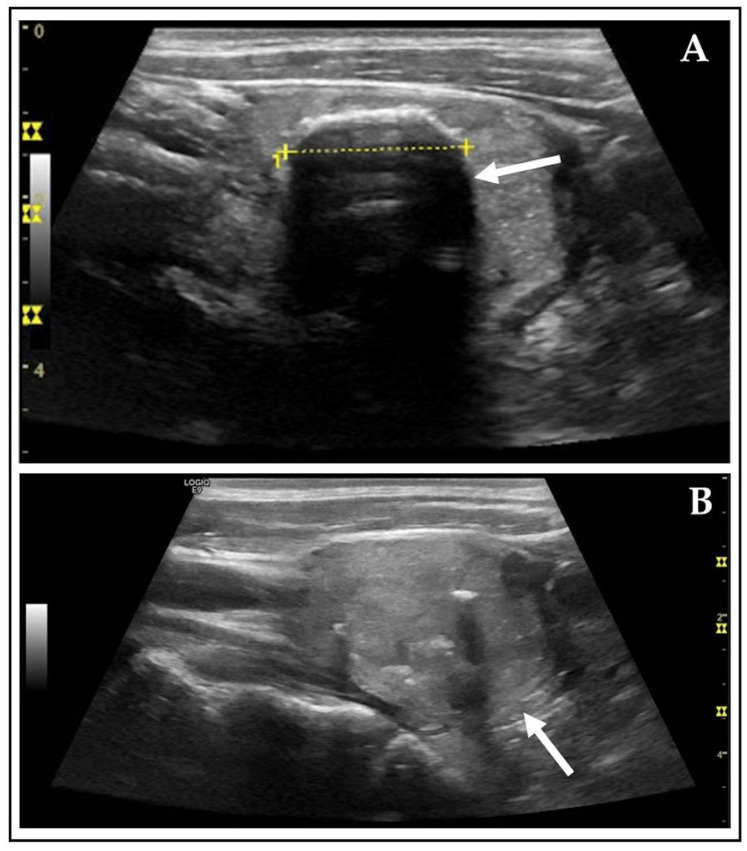
Example for a CL advantage: Evaluation of composition on SIC (**A**) versus CL (**B**). (**A**): Sagittal SIC of AFTN (white arrow) with acoustic shadowing due to peripheral (rim) calcifications, precluding the evaluation of the composition. Measurement lines superimposing AFTN (yellow dotted line) do not capture the full cranio-caudal diameter, relevant for ACR FNC recommendation. (**B**): Sagittal CL image capture of the same AFTN (white arrow), compositional assessment possible.

Acoustic shadowing has been observed to occur with greater frequency on CL in comparison to SIC. Macrocalcifications (~11%) and other factors (~4–6%), such as insufficient gel application, have been identified as contributing causes. In four cases, acoustic shadowing, due to peripheral (rim) calcifications, precluded the evaluation of the composition on SIC. In two of these cases, the evaluation on CL was possible because the artifact did not affect all parts of the AFTN. This highlights one advantage of CL, as they capture the entire TN, allowing for a comprehensive retrospective evaluation on PACS. The following example (Figure 5) illustrates the described phenomenon.

In general, it was observed that CL capture a larger proportion of echogenic foci. All subgroups of this US feature were identified more frequently in comparison to SIC. To elucidate this finding, the ACR TIRADS subgroup “None or large comet-tail artifacts” of the US feature “echogenic foci” was further subdivided into “None” and “Large comet-tail artifacts”. The results of this study indicate that CLs are capable of detecting 12% more echogenic foci in comparison to SIC, influencing the evaluation of Kwak and ACR TIRADS.

The observer encountered greater challenges in evaluating AFTNs on CL (CL versus SIC). Primarily, AFTNs were evaluated on CL with a 3-month interval preceding SIC, which likely resulted in an enhancement of confidence over time among the novice observer. Secondly, the reduced number of images available for evaluation through SIC facilitates contextualizing the given information [15]. Thirdly, the prevalence of artifacts on CL has the potential to impede the evaluation of individual US features, as was discussed above [43,44,45].

The employment of a novice observer in this study proved to be a pivotal element in evaluating the user-friendliness and practical applicability of the investigated RSS. The demonstrated ability of an inexperienced user to effectively apply TIRADS suggests that the method is possibly highly intuitive and easy appliable for less-experienced observers. This finding suggests the possibility of extending the assessment of TIRADS to observers with limited experience. In the clinical routine, there is limited opportunity for extensive training of new employees. Consequently, the evaluation of TIRADS with a minimum training configuration can establish a performance baseline for future distribution of tasks. The findings of this study suggest that an intensive 1 h session with an experienced TIRADS expert, complemented by 15 h of literature research, may suffice to evaluate diagnostic precision. Furthermore, this study firstly demonstrated that US video sequences can be implemented into this approach. 

### Limitations

The investigation utilized a retrospective, unicentric design, which inherently limits the generalizability of the findings. The results may be specific to the patient demographic, equipment and clinical practices of the institution, especially in an iodine-deficient area.

It is important to acknowledge that the present study was subject to a selection bias, including exclusively AFTNs. The observer was cognizant of the outcome, a factor which may have influenced their assessment.

The consistency of the US quality cannot be assured as CL and SIC were recorded by numerous different examiners.

The data assessment was conducted by a single novice observer, which introduces a potential source of observer bias and precludes the examination of interobserver variability. A significant limitation lies in the selection of the trained observer whose talent could have potentially influenced the results. A more experienced observer might have potentially yielded different outcomes. This concern is substantiated by the research of Schenke et al., which concluded that the evaluation of 20 TNs by 12 observers was heavily influenced by the observer’s level of experience [17]. The absence of a comparative study with an experienced examiner precludes any determination regarding the potential influence of the novices experience on the outcomes of this study.

## 5. Conclusions

The present study demonstrated that CLs are a reliable image modality for the assessment of TNs according to RSS. A strong correlation was observed between CL and SIC for both US features and the respective Kwak and ACR TIRADS scores, although the confidence of the novice observer was lower for CL and more artifacts were revealed on CL. Thus, the benefits of CL, particularly the ability to comprehensively monitor the entire organ within the PACS, can be achieved without significant compromise to diagnostic accuracy. Furthermore, in our data, Kwak rating was more consistent with the benign nature of AFTNs than ACR grading. Given that a puncture recommendation according to ACR is available for over 60% of the nodules examined, the results once again emphasize the important role of scintigraphy in the evaluation of TNs.

## Figures and Tables

**Table 1:** Patient characteristics and clinical data.

Category	*n* (%)/Mean ± SD (Median, Range)	Unit
Patient’s demographics		
Total number of patients	407 (100.0)	
Female	257 (63.1)	
Male	150 (36.9)	
Age	62 ± 11 (61, 21–86)	years
Thyroid function		
TSH level	0.2 ± 0.5 (0.1, 0.01–5.2)	mU/L
Euthyroid	93 (22.9)	
Hyperthyroid	313 (76.9)	
Hypothyroid	1 (0.2)	
Thyroid medication		
None	179 (44.0)	
Thyreostatics	89 (21.9)	
L-thyroxine permanently ^1^	10 (2.5)	
L-thyroxine premedication	129 (31.7)	
Ultrasound		
Thyroid volume	31.5 ± 17.1 (27.0, 6.0–122.0)	mL
Number of identified AFTNs	424 (100.0)	
Unifocal	393 (96.6)	
Bifocal	11(2.7)	
Trifocal	3 (0.7)	
Largest diameter of AFTNs		
CL	30 ± 10 (29, 9–62)	mm
SIC	30 ± 10 (30, 9–64)	mm

^1^ paused for scintigraphy. Abbreviations: SD—standard deviation; TSH—thyroid-stimulating hormone; AFTN—autonomously functioning thyroid nodule; CL—cine loops; SIC—static image captures.

**Table 2:** Ultrasound features on CL versus SIC (*n* = 424).

US Features	CL *n* (%)	SIC *n* (%)	Chi^2^ test ^1^	Spearman’s Correlation ^2^
Composition				
Cystic or almost completely cystic	2 (0.5)	0 (0.0)	*p* = 0.499	r_s_ = 0.722 *p* < 0.001
Spongiform	5 (1.2)	2 (0.5)	*p* = 0.451	
Mixed cystic and solid	275 (64.9)	270 (63.7)	*p* = 0.720	
Solid or almost completely solid	140 (33.0)	148 (34.9)	*p* = 0.562	
No assessment possible	2 (0.5)	4 (0.9)	*p* = 0.686	
Echogenicity				
Anechoic	0 (0.0)	0 (0.0)	-	r_s_ = 0.681 *p* < 0.001
Hyperechoic or isoechoic	209 (49.3)	221 (52.1)	*p* = 0.451	
Hypoechoic	205 (48.3)	200 (47.2)	*p* = 0.720	
Very hypoechoic	10 (2.4)	3 (0.7)	*p* = 0.562	
Shape				
Wider-than-tall	362 (85.4)	353 (83.3)	*p* = 0.395	r_s_ = 0.547
Taller-than-wide	62 (14.6)	71 (16.7)	*p* = 0.395	*p* < 0.001
Margin				
Smooth	389 (91.7)	400 (94.3)	*p* = 0.138	r_s_ = 0.483 *p* < 0.001
Ill-defined	35 (8.3)	24 (5.7)	*p* = 0.138	
Lobulated or irregular	0 (0.0)	0 (0.0)	-	
Extra-thyroidal extension	0 (0.0)	0 (0.0)	-	
Echogenic Foci				
None	52 (12.3)	102 (24.1)	*p* < 0.001	r_s_ = 0.603 *p* < 0.001
Large comet-tail artifacts	350 (82.5)	280 (66.0)	*p* < 0.001	
Macrocalcifications	145 (34.2)	109 (25.7)	*p* = 0.007	
Peripheral (rim) calcifications	15 (3.5)	11 (2.6)	*p* = 0.426	
Punctate echogenic foci	41 (9.7)	33 (7.8)	*p* = 0.330	

^1^ *p*-values < 0.05 were considered as significant differences between CL and SIC. ^2^ *p*-values < 0.05 suggests that the correlation (r_s_) was statistically significant and not attributed merely to chance or measurement error (null hypothesis was rejected). Abbreviations: CL—cine loops; SIC—static image captures; US—ultrasound.

**Table 3:** Kwak and ACR TIRADS as well as ACR FNC recommendations (*n* = 424).

TIRADS	CL *n* (%)	SIC *n* (%)	Chi^2^ Test ^1^	Spearman’s Correlation ^2^
Kwak				
3	115 (27.1)	117 (27.6)	*p* = 0.844	r_s_ = 0.699 *p* < 0.001
4A	178 (42.0)	177 (41.7)	*p* = 0.991	
4B	110 (25.9)	102 (24.1)	*p* = 0.552	
4C	19 (4.5)	24 (5.7)	*p* = 0.424	
4C (3 pts.)	19 (4.5)	24 (5.7)	*p* = 0.424	
4C (4 pts.)	0 (0.0)	0 (0.0)	-	
5	0(0.0)	0 (0.0)	-	
No assessment possible ^1^	2 (0.5)	4 (0.9)	*p* = 0.686	
ACR				
TR1	1 (0.2)	0 (0.0)	*p* = 0.999	r_s_ = 0.654 *p* < 0.001
TR2	75 (17.7)	90 (21.2)	*p* = 0.181	
TR3	140 (33.0)	137 (32.3)	*p* = 0.864	
TR4	165 (38.9)	150 (35.4)	*p* = 0.310	
TR4 (4 pts.)	78 (18.4)	74 (17.5)	*p* = 0.769	
TR4 (5 pts.)	36 (8.5)	33 (7.8)	*p* = 0.722	
TR4 (6 pts.)	51 (12.0)	43 (10.1)	*p* = 0.395	
TR5	41 (9.7)	43 (10.1)	*p* = 0.800	
No assessment possible ^1^	2 (0.5)	4 (0.9)	*p* = 0.686	
FNC ACR				
FNC not recommended	85 (20.0)	93 (21.9)	*p* = 0.500	r_s_ = 0.445 *p* < 0.001
FNC recommended	288 (67.9)	271 (63.9)	*p* = 0.218	
Follow up	47 (11.1)	49 (11.6)	*p* = 0.828	
No assessment possible ^3,4^	4 (0.9)	11 (2.6)	*p* = 0.115	

^1^ *p*-values < 0.05 were considered as significant differences between CL and SIC. ^2^ *p*-values < 0.05 suggests that the correlation (r_s_) was statistically significant and not attributed merely to chance or measurement error (null hypothesis was rejected). ^3^ no evaluation of composition. ^4^ no sufficient measurement of diameters possible. Abbreviations: ACR—American College of Radiology; TIRADS—Thyroid Imaging Reporting and Data System; FNC—fine-needle cytology; CL—cine loop, SIC—static image capture.

**Table 4:** US artifacts affecting AFTN (*n* = 424).

Type of Artifacts	CL *n* (%)	SIC *n* (%)	Chi^2^ Test ^1^
Transverse			
None	263 (62.0)	360 (84.9)	*p* < 0.001
Poor image quality	116 (27.4)	24 (5.7)	*p* < 0.001
Acoustic shadowing due to…	64 (15.1)	41 (9.7)	*p* = 0.016
macrocalcifications	44 (10.4)	37 (8.7)	*p* = 0.413
peripheral (rim) calcifications	2 (0.5)	3 (0.7)	*p* = 0.999
other reasons ^2^	18 (4.2)	1 (0.2)	*p* < 0.001
AFTNs incompletely displayed	3 (0.7)	2 (0.5)	*p* = 0.999
Sagittal			
None	247 (58.3)	357 (84.2)	*p* < 0.001
Poor image quality	123 (29.0)	21 (5.0)	*p* < 0.001
Acoustic shadowing due to…	77 (18.2)	49 (11.6)	*p* = 0.007
macrocalcifications	49 (11.6)	43 (10.1)	*p* = 0.508
peripheral (rim) calcifications	3 (0.7)	3 (0.7)	*p* = 0.999
other reasons ^2^	25 (5.9)	3 (0.7)	*p* < 0.001
AFTNs incompletely displayed	73 (17.2)	5 (1.2)	*p* < 0.001
Isthmus	12 (2.8)	1 (0.2)	*p* = 0.003
Upper pole	3 (0.7)	0 (0.0)	*p* = 0.249
Lower pole	39 (9.2)	3 (0.7)	*p* < 0.001
Central	19 (4.5)	1 (0.2)	*p* < 0.001

^1^ *p*-values < 0.05 were considered as significant differences between CL and SIC. ^2^ e.g., insufficient US gel application, misplaced foci. Abbreviations: US—ultrasound; AFTN—autonomously functioning thyroid nodule; CL—cine loops; SIC—static image captures.

**Table 5:** Assessment confidence and related challenging US features (*n* = 424).

Result	CL *n* (%)	SIC *n* (%)	Chi^2^ Test ^1^	Spearman’s Correlation ^2^
Assessment confidence				
1 (Very confident)	168 (39.6)	303 (71.5)	*p* < 0.001	r_s_ = 0.186 *p* < 0.001
2 (Confident)	184 (43.4)	107 (25.2)	*p* < 0.001	
3 (Unsure)	65 (15.3)	14 (3.3)	*p* < 0.001	
4 (Very unsure)	7 (1.7)	0 (0.0)	*p* = 0.015	
US feature with most significant uncertainty				
None	168 (39.6)	303 (71.5)	*p* < *0*.001	r_s_ = 0.133 *p* = 0.006
Composition	77 (18.2)	54 (12.7)	*p* = 0.029	
Echogenicity	20 (4.7)	12 (2.8)	*p* = 0.149	
Shape	12 (2.8)	7 (1.7)	*p* = 0.246	
Margin	32 (7.5)	2 (0.5)	*p* < 0.001	
Echogenic Foci	115 (27.1)	46 (10.8)	*p* < 0.001	

^1^ *p*-values < 0.05 were considered as significant differences between CL and SIC. ^2^ *p*-values < 0.05 suggests that the correlation (r_s_) was statistically significant and not attributed merely to chance or measurement error (null hypothesis was rejected). Abbreviations: US—ultrasound; CL—cine loops; SIC—static image captures.

## Data Availability

The data that support the findings of this study are available from the corresponding author upon reasonable request.

## Abbreviations

The following abbreviations are used in this manuscript:

ACR	American College of Radiology
AFTN	autonomously functioning thyroid nodule
CL	cine loop (ultrasound video sequences)
FNC	fine-needle cytology
PACS	Picture Archiving and Communication System
RSS	Risk stratification systems
SD	Standard deviation
SIC	static image captures (on ultrasound)
SOP	Standard Operation Procedure
T_3_	thyroxin
T_4_	triiodothyronine
TIRADS	Thyroid Imaging Reporting and Data System
TN	thyroid nodule
TS	Tc-99m-pertechnetate thyroid scintigraphy
TSH	thyroid-stimulating hormone
US	ultrasound
